# Fundamental Motor Skills in Children With Specific Learning Disorder

**DOI:** 10.1155/oti/2230534

**Published:** 2026-06-08

**Authors:** Ming-Chih Sung, Chien-Yu Pan, Wen-Fan Chen, Chia-Liang Tsai, Ting-Yi Lu, Chu-Yang Huang

**Affiliations:** ^1^ School of Kinesiology, Rongxiang Xu College of Health and Human Services, California State University, Los Angeles, Los Angeles, California, USA, calstate.edu; ^2^ Department of Physical Education, National Kaohsiung Normal University, Kaohsiung City, Taiwan, nknu.edu.tw; ^3^ Institute of Medical Science and Technology, National Sun Yat-sen University, Kaohsiung City, Taiwan, nsysu.edu.tw; ^4^ Institute of Physical Education, Health and Leisure Studies, National Cheng Kung University, Tainan City, Taiwan, ncku.edu.tw

**Keywords:** learning disability, locomotor skills, motor competence, object control skills, TGMD-2

## Abstract

**Background and Purpose:**

This study investigated fundamental motor skills in children with specific learning disorder (SLD) compared with their typically developing (TD) peers, focusing on individual skill mastery and performance patterns across diagnostic subtypes.

**Methods:**

A total of 90 children (45 SLD and 45 TD) aged 8–11 years participated. Fundamental motor skills were assessed using the process‐oriented Test of Gross Motor Development–2 (TGMD‐2).

**Results:**

Results demonstrated that children with SLD exhibited significantly lower motor skills proficiency than TD peers, with significantly lower scores found across most locomotor (running, galloping, hopping, jumping, and sliding) and object control (batting, catching, throwing, and rolling) skills. The SLD group was significantly less likely to demonstrate mature movement patterns in 9 out of 12 skills, and the majority of children with SLD were categorized in the “Poor” or “Below average” performance ranges, underscoring a critical qualitative deficit in motor execution. However, no significant differences in gross motor performance were observed among the SLD subtypes (dyslexia, dyscalculia, dysorthographia, and mixed).

**Conclusion:**

These findings highlight a pervasive fundamental motor skills deficit that warrants routine motor assessment for children with SLD to inform targeted, process‐oriented interventions aimed at mitigating long‐term developmental risks.

## 1. Introduction

Fundamental motor skills are foundational movements that involve the coordinated combination of various body parts and serve as essential building blocks for more specialized and complex physical activities and sports [1]. These skills are typically categorized into locomotor skills, which involve moving the body from one place to another (e.g., running, hopping, and jumping), and object control skills, which involve manipulating objects (e.g., striking, catching, kicking, and throwing) [2]. Stodden et al. [3] proposed a conceptual model highlighting the interrelationship between physical activity, motor competence, perceived motor skill competence, health‐related physical fitness, and obesity. They emphasized that proficiency in fundamental motor skills during childhood is critical for developing overall motor competence, fostering engagement in diverse physical activities, and promoting an active and healthy lifestyle across the lifespan. Further, evidence has indicated that fundamental motor skills are positively associated with children′s executive function [4, 5], academic achievement [6], and social‐emotional well‐being [7]. As such, promoting the development of fundamental motor skills in early childhood is paramount for fostering holistic child development and long‐term health outcomes.

Specific learning disorder (SLD) is a common neurodevelopmental condition characterized by persistent academic difficulties, including reading, writing, or mathematics, that persist for at least 6 months despite targeted intervention [8]. These difficulties must originate during the school‐age years and cannot be attributed to other developmental, sensory, or psychosocial conditions. The estimated prevalence of SLD ranges from 5% to 15% among school‐aged children, with variation due to differences in diagnostic criteria, assessment methods, and regional factors [9, 10], making it one of the most prevalent developmental disorders in childhood. SLDs are typically identified in specific domains such as reading (dyslexia), writing (dysgraphia), or mathematics (dyscalculia), and may occur independently or co‐occur in combination [8]. Diagnosis is made through a comprehensive evaluation combining clinical, developmental, and educational assessments. Although SLD are primarily recognized based on academic performance, emerging evidence has underscored their impact on broader developmental domains, including executive function [11] and fundamental motor skills [12].

Difficulties with motor skills in children with SLD have been documented in literature, with significant differences observed in motor coordination [13], motor proficiency [14], and fundamental motor skills [15, 16] compared with their typically developing (TD) peers. For instance, Poyraz Findik et al. [17] examined the motor coordination of 57 children with SLD and 30 TD peers, using the Developmental Coordination Disorder Questionnaire and the Purdue Pegboard Test. They found that children with SLD exhibited significantly lower motor performance, particularly in fine motor skills and handwriting, even after controlling for symptoms of attention deficit hyperactivity disorder (ADHD). Similarly, Ibrahim et al. [18] assessed the motor performance of 148 children with SLD using the Movement Assessment Battery for Children‐2 (MABC‐2). Their findings revealed that over half of the children (54.7%) exhibited motor performance difficulties in manual dexterity, whereas 38.5% had definite movement difficulties on the overall MABC‐2 total test score. Further, a recent scoping review conducted by Blanchet and Assaiante [19], which identified 34 relevant studies, confirmed that children and adolescents with SLD generally exhibit poorer fine, gross, and postural motor skills compared with their peers. This review also highlighted that these motor impairments are often exacerbated by the complexity of motor activities and the presence of comorbidities. Collectively, these studies underscore a consistent pattern of motor skill deficits in children with SLD, emphasizing the necessity of early and targeted motor assessments to inform interventions for children with SLD.

Despite the growing body of research, existing studies concerning motor skills deficits among children with SLD often treat SLD as a homogeneous condition [20, 21], overlooking the nuanced differences that may exist between diagnostic subtypes (e.g., dyslexia vs. dyscalculia). Hussein et al. [14] investigated motor performance in 100 children with SLD and 100 of their TD peers aged 9–13, using the Bruininks–Oseretsky Test of Motor Proficiency‐2 (BOT‐2). Their findings revealed that children with SLD scored significantly lower across all BOT‐2 composites and subtests compared with their TD peers. Importantly, their results also highlighted that patterns of motor impairment significantly differed by SLD subtype (dyscalculia, dyslexia, and mixed), with balance issues being the most pronounced in dyslexia, bilateral coordination in dyscalculia, and running speed and agility in mixed‐type SLD. These findings emphasize the need to investigate the unique motor profiles of each SLD subtype in future research to gain a deeper understanding of the specific motor challenges faced by children with different subtypes of SLD in order to inform more targeted intervention strategies.

Research has suggested the importance of adopting a holistic approach to assessing motor skills in children [22, 23]; however, a majority of studies examining motor skills of SLD have relied on broad motor screening tools [17] or focused solely on product‐oriented assessment of motor skills (e.g., BOT‐2 or MABC‐2) [18, 19], with limited emphasis on process‐oriented motor assessments such as the Test of Gross Motor Development–2 (TGMD‐2) [16]. For instance, Baharudin et al. [24] conducted a review of 31 studies that employed standardized movement and/or function assessment tools, including the MABC‐2, BOT‐2 or the pediatric balance scale, and consistently reported motor skill deficits in balance, coordination, and fine motor skills among children with SLD. Yet, their review does not include any process‐oriented assessments (e.g., TGMD‐2). Although product‐oriented tools such as the MABC‐2 and BOT‐2 assess overall motor proficiency, they may not fully capture the qualitative aspects of motor skills that are essential for planning interventions and understanding motor development in this population [24]. Furthermore, among the limited studies that have utilized the TGMD‐2 to examine the motor skills of children with SLD, most have failed to report the individual skill components and mastery levels for each motor skill [15, 21]. Together, these limitations reveal a critical gap in the current literature, which restricts our understanding of the specific movement patterns in children with SLD and, consequently, hinders the development of targeted interventions.

Given the increasing recognition of motor skill challenges in children with SLD, there is a pressing need for assessments that move beyond overall performance scores to capture the qualitative aspects of movement and provide a more detailed understanding of how individual fundamental motor skills are performed. To the best of the authors′ knowledge, no research to date has simultaneously employed a process‐oriented motor skills assessment such as the TGMD‐2 while also reporting individual skill components and mastery levels of fundamental motor skills in children with SLD. By addressing this crucial gap in the literature, our study has highlighted the identification of both overall fundamental motor skills and specific movement patterns that require the most instructional support, particularly as they may vary across different subtypes of SLD. Therefore, the purpose of this study was to examine fundamental motor skills in children diagnosed with SLD using the TGMD‐2 with a specific focus on individual skill mastery and performance patterns across diagnostic subtypes (i.e., dyslexia, dyscalculia, dysorthographia, and mixed‐type SLD).

## 2. Methods

### 2.1. Ethical Approval and Consent to Participate

This study was approved by the National Cheng Kung University Human Research Ethics Committee (Approval No.: NCKU HREC‐F‐111‐074‐2). Prior to participation, all participants and their parents were informed about the study and its objectives, and informed consents were signed and obtained from all participants and their parents or legal guardians.

### 2.2. Participants

Forty‐five children with SLD (13 girls and 32 boys), aged between 8 and 11 years old in the year of enrollment, participated in this cross‐sectional study. They were recruited from four mainstream elementary schools in an urban area of Taiwan. To determine the performance level of children with SLD, 45 TD peers (13 girls and 32 boys), attending the same schools, were recruited according to the age and gender distribution of the children with SLD in the present study to provide reference values. For each child, the individual school files containing information about child characteristics (i.e., age, gender, type of SLD) were reported by school teachers, and these children were reported having a normal intelligence quotient. To be diagnosed with an SLD, a person must meet the following criteria: (1) have difficulties in at least one of the following areas for at least one semester (around 6 months) despite targeted help, such as reading, understanding, spelling, written expression, calculation, and mathematical reasoning; (2) have academic skills that are substantially below what is expected for the child′s age and cause problems in school, work or everyday activities; and (3) learning difficulties are not due to other conditions, such as intellectual disability, vision or hearing problems, a neurological condition (e.g., pediatric stroke), adverse conditions such as economic or environmental disadvantage, lack of instruction, or difficulties speaking/understanding the language. Within the SLD group, children were classified into one of four subtypes: dyslexia, dyscalculia, dysorthographia, or mixed type, based on their clinical diagnosis.

### 2.3. Measurements

Fundamental motor skills were assessed using the TGMD‐2 [2]. The TGMD‐2 is a standardized assessment that combines norm‐referenced and criterion‐referenced approaches to evaluate 12 fundamental motor skills in children aged 3–10 years and 11 months. These skills are categorized into two subtests: locomotor skills (run, gallop, hop, leap, horizontal jump, and slide) and object control skills (stationary dribble, catch, kick, strike a stationary ball, overhand throw, and underhand roll). Each skill consists of 3–5 qualitative performance criteria. Each criterion is scored dichotomously (1 = present, 0 = absent), allowing for an objective evaluation of skill components. A perfect raw score on each subtest is 24 points, resulting in a combined maximum of 48 points across both subtests.

All participants performed the 12 skills during a single testing session, and performances were video‐recorded using a SONY Mini DV Digital Handycam (HDR‐SR12) to ensure accurate scoring. Administration adhered to the standardized protocol described in the TGMD‐2 manual [2]. Each child attempted each skill twice, and scores were summed across trials. For example, a skill with four criteria could yield a raw score ranging from 0 to 8. Raw scores for each subtest were converted to age‐adjusted standard scores (range: 1–20) and combined to compute the gross motor quotient, which provides an overall index of gross motor proficiency with scores ranging from 46 to 160. Subtest standard scores and the overall gross motor quotient were used to evaluate each child′s motor proficiency relative to the standardized norms provided in the TGMD‐2 manual.

The TGMD‐2 has demonstrated strong psychometric properties. Previous studies have reported high internal consistency, test–retest reliability, and interrater reliability, with coefficients exceeding 0.85 for locomotor and object control subtests, and the gross motor development quotient (GMDQ) [2,25]. Evidence also supports its content validity, criterion‐related validity, and construct validity as an assessment of fundamental motor skills in children [2]. Research has further demonstrated strong reliability (*α* > 0.90) and validity for diverse populations [26], including children with disabilities [27].

### 2.4. Procedure

The TGMD‐2 was administered in a gymnasium to provide adequate space and a controlled environment for the assessment. Prior to data collection, the primary investigator and research assistants completed training on the administration and scoring procedures outlined in the TGMD‐2 manual. The testing area was arranged according to standardized TGMD‐2 guidelines, with clear markings for each skill station and sufficient safety space for movement. Each child was assessed individually during a single session lasting approximately 20–40 min. Before beginning the formal trials, participants were given verbal instructions and a visual demonstration of each skill by the examiner. Children were allowed one practice trial followed by two formal test trials for each skill. All performances were video‐recorded using a SONY Mini DV Digital Handycam (HDR‐SR12) positioned at a 45° angle to the side of the participant to capture full‐body movement. To ensure the safety and optimal performance of the participants, a test administrator was present throughout the entire session to provide positive encouragement and verbal cues as needed while maintaining the standardized protocol.

Scoring was conducted by two independent raters who were trained by the principal investigator. Training sessions involved two elementary school‐aged children demonstrating the TGMD‐2 skills, during which the raters were guided to identify strengths and weaknesses in performance. The principal investigator and raters engaged in detailed discussions to clarify the specific performance criteria for each skill. Following training, interrater reliability was established prior to scoring the full dataset by having both raters independently score a randomly selected 20% subset of the videos. To ensure consistency, a monitoring system was implemented to maintain interrater agreement above 0.85.

### 2.5. Statistical Analysis

Descriptive statistics were calculated to summarize participant characteristics (e.g., age, body mass index [BMI], and gender) and the distribution of diagnostic subtypes within the SLD group. Group differences in demographic variables were examined using independent *t*‐tests for continuous variables (i.e., age and BMI). To analyze performance on the TGMD‐2, the frequency of children meeting mature performance criteria for each skill was compared between the SLD and TD groups using chi‐square tests. Analysis of covariance (ANCOVA) was conducted to compare TGMD‐2 raw scores between the SLD and TD groups while statistically controlling for age and gender. Separate ANCOVAs were performed for locomotor subtests, object control subtests, and individual skill scores. Tukey′s post hoc test was examined if significant group difference was observed. Finally, chi‐square analyses were also applied to compare the distribution of TGMD‐2 performance categories (e.g., “very poor,” “poor,” “below average,” “average,” and “above average”) between the SLD and TD groups, as well as across SLD diagnostic subtypes (dyslexia, dyscalculia, dysorthographia, and mixed). Effect sizes were reported using partial eta squared ($\eta_{p}^{2}$) for ANCOVA analyses and Cramér′s V for chi‐square analyses. Effect sizes were interpreted based on commonly used guidelines ($\eta_{p}^{2}$: 0.01 = small, 0.06 = moderate, and 0.14 = large; Cramér′s V: 0.10 = small, 0.30 = moderate, and 0.50 = large). All statistical analyses were conducted using SPSS (Version 18.0) for Windows (SPSS Inc. Chicago, Illinois, United States), with an alpha level of 0.05 set for significance.

## 3. Results

### 3.1. Participant Characteristics

The present study included 45 children with SLD and 45 age‐ and gender‐matched TD children, with both groups consisting of 71% boys. On average, children with SLD were significantly older than their TD peers by 1 year (10.44 ± 0.48 vs. 9.34 ± 0.54 years; *t* = 10.14, *p* < 0.001). However, no significant differences in BMI were observed between the groups. Within the SLD group, the distribution of diagnoses was as follows: dyslexia (57.8%), dyscalculia (8.9%), dysorthographia (4.4%), and mixed‐type diagnoses (28.9%). Participant characteristics are presented in Table 1.

**Table 1:** Participant demographics.

Variable	SLD (*n* = 45)	TD (*n* = 45)	*t*	*p*
	*M* ± *S* *D*	*M* ± *S* *D*		
Age (years)	10.44 ± 0.48	9.34 ± 0.54	10.14	< 0.001
BMI (kg/m^2^)	19.31 ± 3.93	18.39 ± 3.23	1.22	0.227
Gender, *n* (%)				
Girls	13 (28.9%)	13 (28.9%)		
Boys	32 (71.1%)	32 (71.1%)		
Type of SLD, *n* (%)				
Dyslexia	26 (57.8%)	—		
Dyscalculia	4 (8.9%)	—		
Dysorthographia	2 (4.4%)	—		
Mixed	13 (28.9%)	—		

Abbreviations: BMI, body mass index; *M* ± SD, mean ± standard deviation; SLD, specific learning disability; TD, typical development.

### 3.2. Fundamental Motor Skills Performance (TGMD‐2 Skill)

The frequency of the mature performance characteristics defined by the TGMD‐2 was calculated for each fundamental motor skill. Table 2 displays the proportion of children with SLD and their TD peers who met all criteria for each skill. Overall, children with SLD were significantly less likely to demonstrate mature movement patterns in 9 out of the 12 TGMD‐2 skills. Within the locomotor skills, significant group differences were found in running (*χ* ^2^ [5, n = 90] = 31.61, *p* < 0.001, Cramér′s *V* = 0.593), galloping (*χ* ^2^ [5, *n* = 90] = 73.32, *p* < 0.001, Cramér′s *V* = 0.903), hopping (*χ* ^2^ [8, *n* = 90] = 32.50, *p* < 0.001, Cramér′s *V* = 0.601), jumping (*χ* ^2^ [6, *n* = 90] = 24.30, *p* < 0.001, Cramér′s *V* = 0.520), and sliding (*χ* ^2^ [6, *n* = 90] = 18, *p* = 0.006, Cramér′s *V* = 0.447). For object control skills, significant differences were observed in batting (*χ* ^2^ [6, *n* = 90] = 18.17, *p* = 0.006, Cramér′s *V* = 0.449), catching (*χ* ^2^ [2, *n* = 90) = 12.80, *p* = 0.002, Cramér′s *V* = 0.377), throwing (*χ* ^2^ [8, *n* = 90] = 27.46, *p* = 0.001, Cramér′s *V* = 0.552), and rolling (*χ* ^2^ [7, *n* = 90] = 20.62, *p* = 0.004, Cramér′s *V* = 0.479). No significant differences emerged for leaping (*p* = 0.733, Cramér′s *V* = 0.083), dribbling (*p* = 0.461, Cramér′s *V* = 0.293), and kicking (*p* = 0.546, Cramér′s *V* = 0.185). Despite the lack of statistical significance, the observed effect sizes for these three motor skills suggest that small to moderate group differences may still exist but were not detected in the present analyses.

**Table 2:** Number and percentage of each group who mastered each skill.

	SLD (*n* = 45)	TD (*n* = 45)	*χ* ^2^	*p*	Cramér′s *V*
Locomotor					
Run (*n*, %)	17 (37.8)	42 (93.3)	31.61	< 0.001	0.593
Gallop (*n*, %)	1 (2.2)	39 (86.7)	73.32	< 0.001	0.903
Hop (*n*, %)	1 (2.2)	11 (24.4)	32.50	< 0.001	0.601
Leap (*n*, %)	33 (73.3)	36 (80.0)	0.62	0.733	0.083
Jump (*n*, %)	7 (15.6)	21 (46.7)	24.30	< 0.001	0.520
Slide (*n*, %)	30 (66.7)	45 (100)	18.00	0.006	0.447
Object control					
Bat (*n*, %)	12 (26.7)	25 (55.6)	18.17	0.006	0.449
Dribble (*n*, %)	23 (51.1)	31 (68.9)	7.72	0.461	0.293
Catch (*n*, %)	28 (62.2)	42 (93.3)	12.80	0.002	0.377
Kick (*n*, %)	15 (33.3)	9 (20.0)	3.07	0.546	0.185
Throw (*n*, %)	12 (26.7)	27 (60)	27.46	0.001	0.552
Roll (*n*, %)	7 (15.6)	21 (46.7)	20.62	0.004	0.479

*Note:* Cramér′s V = effect size for chi‐square analyses.

Abbreviations: SLD, specific learning disability; TD = typical development.

### 3.3. Group Comparisons on the TGMD‐2 Skill Performance

The results of the ANCOVA, with age and gender as covariates, revealed significant differences in locomotor and object control skills between children with SLD and their TD peers. For locomotor skills, the SLD group exhibited significant lower scores across all assessed activities but leaping, including locomotor (*M* = 36.38 vs. *M* = 44.69, *p* < 0.001, $\eta_{p}^{2}=0.465$), running (*M* = 6.69 vs. *M* = 7.91, *p* < 0.001, $\eta_{p}^{2}=0.184$), galloping (*M* = 5.51 vs. *M* = 7.82, *p* < 0.001, $\eta_{p}^{2}=0.448$), hopping (*M* = 5.98 vs. *M* = 8.16, *p* < 0.001, $\eta_{p}^{2}=0.147$), jumping (*M* = 5.47 vs. *M* = 7.11, *p* < 0.001, $\eta_{p}^{2}=0.263$), and sliding (*M* = 7.13 vs. *M* = 8.00, *p* < 0.001, $\eta_{p}^{2}=0.157$). Leaping was not significantly different between groups (*p* = 0.948, $\eta_{p}^{2}=0.000$). These findings indicate moderate to large effect sizes for most locomotor skills. For object control skills, significant differences were observed in batting (*M* = 7.76 vs. *M* = 9.33, *p* = 0.010, $\eta_{p}^{2}=0.074$), throwing (*M* = 5.29 vs. *M* = 7.31, *p* = 0.002, $\eta_{p}^{2}=0.105$), and rolling (*M* = 5.56 vs. *M* = 7.00, *p* < 0.001, $\eta_{p}^{2}=0.229$), with the SLD group generally demonstrating lower performance. These differences reflected small to moderate effect sizes. However, dribbling (*p* = 0.122, $\eta_{p}^{2}=0.028$), catching (*p* = 0.052, $\eta_{p}^{2}=0.043$), and kicking (*p* = 0.092, $\eta_{p}^{2}=0.033$) were not significantly different between the two groups, with small effect sizes observed (see Table 3).

**Table 3:** Group comparisons on locomotor and object control skills.

	SLD (*n* = 45)		TD (*n* = 45)		*F*_(1, 86)_	*p*	$\eta_{p}^{2}$
	*M*	SD	*M*	SD			
Locomotor	36.38 (28–46)	4.41	44.69 (38–48)	2.38	74.86	< 0.001	0.465
Run (8)	6.69 (2–8)	1.35	7.91 (6–8)	0.36	19.39	< 0.001	0.184
Gallop (8)	5.51 (2–8)	1.06	7.82 (6–8)	0.49	69.66	< 0.001	0.448
Hop (10)	5.98 (0–10)	1.80	8.16 (3–10)	1.57	14.87	< 0.001	0.147
Leap (6)	5.60 (4–6)	0.72	5.69 (4–6)	0.67	0.00	0.948	0.000
Jump (8)	5.47 (1–8)	1.75	7.11 (3–8)	1.09	30.63	< 0.001	0.263
Slide (8)	7.13 (1–8)	1.65	8.00 (8–8)	0.00	16.02	< 0.001	0.157
Object control	37.07 (24–48)	5.10	43.27 (36–47)	2.76	21.86	< 0.001	0.203
Bat (10)	7.76 (3–10)	2.22	9.33 (6–10)	0.95	6.88	0.010	0.074
Dribble (8)	6.29 (0–8)	2.40	7.36 (4–8)	1.11	2.45	0.122	0.028
Catch (6)	5.58 (4–6)	0.58	5.93 (5–6)	0.25	3.89	0.052	0.043
Kick (8)	6.60 (4–8)	1.21	6.33 (4–8)	1.04	2.91	0.092	0.033
Throw (8)	5.29 (0–8)	2.41	7.31 (4–8)	0.97	10.08	0.002	0.105
Roll (8)	5.56 (1–8)	1.67	7.00 (4–8)	1.19	25.50	< 0.001	0.229

*Note:* Group comparisons use ANCOVA with age and gender as covariates; the numerals in the brackets next to each skill indicate maximum skill criterion per skill.

Abbreviations: $\eta_{p}^{2}$, partial eta squared (effect size); *M*, mean; *SD*, standard deviation; (range of scores); SLD, specific learning disability; TD, typical development.

Performance categories derived from the TGMD‐2 revealed significant group differences in locomotor skills, object control skills, and gross motor quotient. For locomotor performance, the distribution differed significantly between groups, *χ* ^2^ = 48.99, *p* < 0.001, Cramér′s *V* = 0.738. A majority of TD children were rated as “Average” (64.4%) or “Above average” (31.1%), whereas none fell into the “Poor” or “Very poor” categories. In contrast, most children with SLD were categorized as “Poor” (33.3%) or “Below average” (33.3%), with no child classified as “Above average.” A similar pattern was observed for object control performance, *χ* ^2^ = 30.33, *p* < 0.001, Cramér′s *V* = 0.581. The majority of TD children fell in the “Average” category (77.8%), whereas children with SLD were more evenly distributed across lower performance categories, including “Very poor” (13.3%), “Poor” (28.9%), and “Below average” (28.9%). For gross motor quotient, group differences were again significant, *χ* ^2^ = 43.76, *p* < 0.001, Cramér′s *V* = 0.697. Most TD children were rated as “Average” (80.0%) or “Above average” (6.7%), whereas the SLD group was primarily classified as “Very poor” (28.9%), “Poor” (26.7%), or “Below average” (24.4%), with no participants achieving an “Above average” rating (see Table 4).

**Table 4:** Group comparisons on category of performance.

	SLD	TD	*χ* ^2^	*p*	Cramér′s *V*
Locomotor			48.99	< 0.001	0.738
Very poor	3 (6.7%)	0 (0%)			
Poor	15 (33.3%)	0 (0%)			
Below average	15 (33.3%)	2 (4.4%)			
Average	12 (26.7%)	29 (64.4%)			
Above average	0 (0%)	14 (31.1%)			
Object control			30.33	< 0.001	0.581
Very poor	6 (13.3%)	0 (0%)			
Poor	13 (28.9%)	1 (2.2%)			
Below average	13 (28.9%)	8 (17.8%)			
Average	11 (24.4%)	35 (77.8%)			
Above average	2 (4.4%)	1 (2.2%)			
Gross motor quotient			43.76	< 0.001	0.697
Very poor	13 (28.9%)	0 (0%)			
Poor	12 (26.7%)	1 (2.2%)			
Below average	11 (24.4%)	5 (11.1%)			
Average	9 (20.2%)	36 (80.0%)			
Above average	0 (0%)	3 (6.7%)			

*Note:* Cramér′s V = effect size for chi‐square analyses.

Abbreviations: SLD, specific learning disability; TD, typical development.

### 3.4. TGMD‐2 Skill Performance Across SLD Subtypes

Chi‐square analyses examining the association between TGMD‐2 subtests of locomotor skills, object control skills, and gross motor quotient and different subtypes of SLD, including dyslexia, dyscalculia, dysgraphia, and mixed types, revealed no significant differences. For locomotor skills (*χ* ^2^ = 8.09, *p* = 0.525, Cramér′s *V* = 0.245), the distribution showed similar patterns across groups, with dyslexia having 42.3% “below average” and 30.8% “average,” dyscalculia with 25% in each category, dysgraphia with 50% “poor” and 50% “below average,” and the mixed group with 53.8% “poor.” In object control skills (*χ* ^2^ = 10.87, *p* = 0.540, Cramér′s *V* = 0.284), dyslexia had 30.8% in both “poor” and “below average,” mixed types had 30.8% “very poor” and 30.8% “poor,” dyscalculia had 50% “below average,” and dysgraphia had 50% in both “below average” and “average.” For gross motor quotient (*χ* ^2^ = 12.16, *p* = 0.204, Cramér′s *V* = 0.300), the mixed group had the highest proportion of “very poor” (46.2%), followed by dyslexia (23.1%), and dysorthographia fell into the “poor” category, whereas dyscalculia showed more variability with each category (see Table 5). Although these associations were not statistically significant, the effect sizes ranged from small to moderate, suggesting potentially meaningful differences across SLD subtypes that may not have been detected due to limited sample size.

**Table 5:** Association of TGMD‐2 subtests of locomotor and object control skills and gross motor quotient among children with SLD.

	Types						
	Dyslexia	Dyscalculia	Dysorthographia	Mixed	*χ* ^2^	*p*	Cramér′s *V*
Locomotor					8.09	0.525	0.245
Very poor	1 (3.8%)	1 (25%)	0 (0%)	1 (7.7%)			
Poor	6 (23.1%)	1 (25%)	1 (50%)	7 (53.8%)			
Below average	11 (42.3%)	1 (25%)	1 (50%)	2 (15.4%)			
Average	8 (30.8%)	1 (25%)	0 (0%)	3 (23.1%)			
Object control					10.87	0.540	0.284
Very poor	1 (3.8%)	1 (25%)	0 (0%)	4 (30.8%)			
Poor	8 (30.8%)	1 (25%)	0 (0%)	4 (30.8%)			
Below average	8 (30.8%)	2 (50%)	1 (50%)	2 (15.4%)			
Average	7 (26.9%)	0 (0%)	1 (50%)	3 (23.1%)			
Above average	2 (7.7%)	0 (0%)	0 (0%)	0 (0%)			
Gross motor quotient					12.16	0.204	0.300
Very poor	6 (23.1%)	1 (25%)	0 (0%)	6 (46.2%)			
Poor	5 (19.2%)	2 (50%)	2 (100%)	3 (23.1%)			
Below average	9 (34.6%)	1 (25%)	0 (0%)	1 (7.7%)			
Average	6 (23.1%)	0 (0%)	0 (0%)	3 (23.1%)			

*Note:* Cramér′s V = effect size for chi‐square analyses.

Abbreviations: SLD, specific learning disability; TGMD‐2, Test of Gross Motor Development‐2.

## 4. Discussion

The primary aim of this study was to investigate the proficiency of fundamental motor skills in children with SLD using the process‐oriented assessment (i.e., TGMD‐2), as compared with their TD peers, with an emphasis on specific skill mastery and performance patterns among different diagnostic subtypes (i.e., dyslexia, dyscalculia, dysorthographia, and mixed‐type SLD). The present study found that children with SLD exhibited significantly lower mature movement patterns in most TGMD‐2 skills. Furthermore, children with SLD demonstrated significantly lower scores in overall locomotor and object control skills and were more frequently categorized into “poor” or “below average” performance categories for locomotor skills, object control skills, and gross motor quotient compared with their TD peers. However, no significant differences in motor skill performance were observed across different SLD subtypes.

The findings that children with SLD demonstrated significantly lower levels of fundamental motor skills, both overall and across specific skills, than their TD peers are in line with previous studies [16, 19, 28]. Westendorp et al. [16] studied 144 children with SLD in primary special‐needs schools and observed that these children scored considerably lower in both locomotor and object control skills than TD children. Similar trends have been observed in studies utilizing different motor skills assessments (e.g., MABC‐2 or BOT‐2), where children with SLD consistently exhibit lower motor skills performance compared with their TD peers [14, 17, 18]. One plausible explanation for this consistent finding is the cerebellar deficit hypothesis, which suggests that structural or functional abnormalities in the cerebellum, a brain region essential for procedural learning and automatization, contribute to both reading difficulties and motor impairments observed in individuals with SLD [13, 29]. Supporting this hypothesis, neuroimaging studies have identified atypical activation patterns within cerebellar regions among individuals with dyslexia compared with TD readers [30]. Given the cerebellum′s role in timing, sequencing, and coordination, its dysfunction may hinder the development of smooth and efficient motor execution [31].

Although a substantial body of literature indicates motor skill deficits in children with SLD, the findings of Getchell et al. [15] present an interesting counterpoint. Their study, which also utilized the TGMD‐2 to assess children with dyslexia, reported no significant differences in either locomotor skills or object control subtests between children with dyslexia and their TD peers. A crucial factor potentially contributing to this divergent finding lies in the educational environment from which Getchell et al.′s participants were recruited. Unlike many public‐school settings, the schools attended by the dyslexic participants provided extensive physical education curricula, including dedicated practice time ranging from three to five times per week, along with opportunities for individualized special instruction. Getchell et al. [15] posited that this enriched exposure to motor skill development and targeted support may have mitigated the motor difficulties often observed in this population. This suggests that increased opportunities for physical activity and specialized motor skill instruction could play a significant role in improving motor outcomes for children with SLD.

Our analysis of skill mastery revealed that the SLD group was significantly less likely to meet all performance criteria for 9 out of 12 skills, a finding that strongly supports the notion that the deficit is qualitative (i.e., process‐oriented) rather than merely quantitative (i.e., product‐oriented). This result corroborates the findings reported by Nonis and Jernice [32] in children with mild learning disabilities, who performed significantly lower on 8 of the 12 TGMD‐2 items, including gallop, hop, leap, jump, slide, strike, dribble, and underhand roll, as compared with the TGMD‐2 norm. Our observation that deficits were pervasive across nearly all locomotor (running, galloping, hopping, jumping, and sliding) and object control (batting, catching, throwing, and rolling) skills highlights the generalized nature of the motor challenges in children with SLD. This widespread deficit in fundamental motor skills suggests that basic motor competency itself is compromised in this population. The greater difficulty observed in mastering most skills may stem from broader issues in sensorimotor integration and in the internal representations needed for planning and executing movements [19]. Such challenges likely reflect an impaired ability to create and update accurate internal models of action [19]. Importantly, performance on the TGMD‐2 may not solely reflect motor execution but also the cognitive processes that support movement. The assessment requires children to observe, interpret, and reproduce movement patterns following verbal and visual demonstrations, thereby placing demands on attention, working memory, and sequencing abilities [33–35]. As such, lower performance among children with SLD may partially reflect challenges in these cognitive domains, in addition to previously discussed difficulties in sensorimotor integration and movement planning, rather than representing purely motor execution deficits. This cognitive–motor interaction may be particularly relevant for children with mixed SLD presentations, where multiple domains of impairment coexist. Recognizing this interface provides a more comprehensive interpretation of motor performance in this population.

The overwhelming majority of children with SLD in our study were classified in the “Below average,” “Poor,” or “Very poor” ranges for locomotor skills, object control skills, and the overall gross motor quotient as compared with their TD peers. This finding resonates with the broader developmental framework proposed by Stodden and colleagues [3], which conceptualizes a reciprocal developmental relationship between motor competence and physical activity participation. According to this model, children with low motor competence are less likely to engage in physical activity, which in turn limits opportunities for practice and further skill refinement. Over time, this reduced participation reinforces the motor deficit and contributes to a downward developmental trajectory. Children with SLD classified in the “Poor” or “Very poor” categories are therefore significantly constrained in their ability to participate confidently and successfully in complex and organized physical activities with their peers. This subsequent low participation further leads to reduced opportunities for practice, reinforcing the motor deficit and widening the gap over time. Longitudinal evidence indicates that children with SLD exhibit a performance lag of at least three to 4 years in fundamental motor skills compared with their TD peers, a trajectory that is likely initiated by this lower performance profile [21]. This consistent classification profile should serve as a strong indicator that motor assessment is a crucial, but often underestimated, clinical practice in the evaluation of SLD [17].

Our results indicated that there were no significant differences in fundamental motor skill (i.e., both locomotor and object control) performance among the dyslexia, dyscalculia, dysorthographia, and mixed groups. This finding is contrary to the work of Hussein et al. [14], who employed BOT‐2 to investigate motor performance across different types of SLD. Their findings indicated that balance is affected in dyslexia, bilateral coordination in dyscalculia, and running speed and agility in the mixed group. A possible explanation for these divergent findings lies in the differences between the assessment tools and the motor domains they prioritize. Our study utilized the TGMD‐2, which is a process‐oriented assessment focused qualitatively on fundamental, gross motor pattern maturity. In contrast, the BOT‐2 is a product‐oriented and quantitative instrument that includes subtests focused on fine motor, balance, coordination, and speed‐based skills. The ability to detect specific subtype differences appears to be greater when measuring the discrete, complex components found in the BOT‐2, which require high levels of executive control. Given that the TGMD‐2 measures a generalized fundamental motor skill ability, the lack of distinction across subtypes suggests the presence of a global motor deficit common to all SLD categories.

Building upon these methodological considerations, differences in sample characteristics, particularly in sample size and group distribution, may also account for the inconsistent findings across studies. Hussein et al.′s [14] larger, yet uneven, sample (58 dyscalculia, 22 dyslexia, and 20 mixed) contrasts with our smaller, more limited sample (26 dyslexia, 4 dyscalculia, 2 dysorthographia, and 13 mixed). A smaller sample, especially when distributed across multiple subgroups, inherently reduces the statistical power to detect true differences between groups [36]. Beyond methodological explanations, the lack of distinction in gross motor skills across subtypes of SLD may also be interpreted through the lens of the atypical brain development framework [12, 37], which posits that neurodevelopmental disorders share overlapping neural vulnerabilities rather than distinct, domain‐specific pathways. This interpretation is reinforced by the high prevalence of co‐occurring conditions such as developmental coordination disorder and ADHD in children with SLD [17], both of which are closely associated with generalized motor impairments. Consequently, the shared genetic or neurological influences related to these common comorbidities may overshadow any subtle motor profile differences that might exist between dyslexia, dyscalculia, and dysorthographia.

Several limitations of this study should be acknowledged. First, the relatively small sample size and uneven distribution across SLD subtypes limited the statistical power to detect potential differences among diagnostic groups. Future research with larger and more balanced samples is warranted to clarify whether distinct motor profiles exist across subtypes of SLD. Second, information on comorbid conditions such as ADHD or Developmental Coordination Disorder was not available. Given the high comorbidity rates between these disorders and SLD, future studies should control for or examine the interactive effects of these co‐occurring conditions to better isolate the motor difficulties specifically attributable to SLD. Third, because the TGMD‐2 primarily assesses gross motor performance, the present findings may not fully capture fine motor or balance‐related deficits that are also prevalent in children with SLD. Although the TGMD‐2 provides a detailed process‐oriented evaluation of gross motor skills, it does not assess fine motor abilities such as manual dexterity and graphomotor coordination. This limitation is particularly relevant given the inclusion of dysorthographia within the SLD subtypes, as this condition is often associated with deficits in fine motor control and handwriting‐related skills. As such, the absence of fine motor assessment in the present study may have obscured potential subtype‐specific differences. Future research should incorporate comprehensive motor batteries that include both gross and fine motor domains to provide a more comprehensive understanding of motor functioning in children with SLD. Lastly, the cross‐sectional design limits causal interpretations. Longitudinal studies are needed to examine how motor skills evolve over time and its influence on academic, social, and physical outcomes.

The results of this study have several important practical implications for clinicians, educators, and researchers serving children with SLD. First, the pervasive and poor categorization of fundamental motor skills mastery in the SLD group reinforces the necessity of universal motor skills assessment as a routine component of the comprehensive evaluation process, moving beyond the academic domain. Second, the clear evidence of poor skill mastery across nearly all locomotor and object control skills highlights the need for process‐oriented intervention. Therapy and physical education instruction should focus explicitly on correcting the component patterns of movement (e.g., proper opposition during running, correct weight transfer during throwing), rather than solely focusing on the outcome (e.g., distance thrown, speed achieved). Third, our observation that the motor skills deficit is largely shared across SLD subtypes indicates that motor skills intervention that integrate skill‐specific instruction can be broadly implemented for children across dyslexia, dyscalculia, dysorthographia, and mixed diagnoses, focusing on the common deficits in gross motor mastery.

## 5. Conclusion

This study provides clear evidence that children with SLD exhibit generalized deficits in fundamental motor skills compared with their TD peers. These deficits were evident across both locomotor and object control domains and were consistent across SLD subtypes. The majority of children with SLD were categorized as having “Poor” or “Below average” fundamental motor skills, highlighting a pervasive delay in motor competence that may limit their participation in physical and social activities and further restrict opportunities for skill refinement. By emphasizing process‐oriented evaluation through the TGMD‐2, this study contributes to a more nuanced understanding of qualitative aspects of motor performance in SLD. Future research should continue to explore the neural and environmental mechanisms underlying these motor deficits and develop evidence‐based interventions that strengthen motor competence as a pathway toward enhancing academic success, physical activity engagement, and overall quality of life in children with SLD.

## Funding

No funding was received for this manuscript.

## Ethics Statement

This study was approved by the National Cheng Kung University Human Research Ethics Committee (Approval No.: NCKU HREC‐F‐111‐074‐2). Prior to participation, all participants and their parents were informed about the study and its objectives, and the informed consents were signed and obtained from all participants and their parents or legal guardians.

## Conflicts of Interest

The authors declare no conflicts of interest.

## Data Availability

The data that support the findings of this study are available on request from the corresponding author. The data are not publicly available due to privacy or ethical restrictions.

## References

[1] Lubans D. R. , Morgan P. J. , Cliff D. P. , Barnett L. M. , and Okely A. D. , Fundamental Movement Skills in Children and Adolescents, Sports Medicine. (2010) 40, no. 12, 1019–1035, 10.2165/11536850-000000000-00000. 21058749

[2] Ulrich D. A. , Test of Gross Motor Development, 2000, 2nd edition, PRO-ED.

[3] Stodden D. F. , Goodway J. D. , Langendorfer S. J. , Roberton M. A. , Rudisill M. E. , Garcia C. , and Garcia L. E. , A Developmental Perspective on the Role of Motor Skill Competence in Physical Activity: An Emergent Relationship, Quest. (2008) 60, no. 2, 290–306, 10.1080/00336297.2008.10483582.

[4] Pan C. Y. , Sung M. C. , Tsai C. L. , Chen F. C. , Chen Y. J. , and Chen C. C. , The Relationships Between Motor Skills and Executive Functions in Children With and Without Autism Spectrum Disorder, Autism Research. (2024) 17, no. 6, 1149–1160, 10.1002/aur.3136, 38641916. 38641916

[5] Sung M. C. , McClelland M. M. , Massey W. , Logan S. W. , and Mac Donald M. , Association Between Motor Skills and Executive Function of Children With Autism Spectrum Disorder in Taiwan and the United States, Frontiers in Public Health. (2024) 11, 1292695, 10.3389/fpubh.2023.1292695, 38249390. 38249390 PMC10796658

[6] Greenburg J. E. , Carlson A. G. , Kim H. , Curby T. W. , and Winsler A. , Early Visual-Spatial Integration Skills Predict Elementary School Achievement Among Low-Income, Ethnically Diverse Children, Early Education and Development. (2020) 31, no. 2, 304–322, 10.1080/10409289.2019.1636353.

[7] Eliassy M. , Khajavi D. , Shahrjerdi S. , and Mirmoezzi M. , Associations Between Physical Activity and Gross Motor Skills With Social Development in Children With Learning Disabilities, International Journal of Sport Studies for Health. (2021) 4, no. 1, 10.61838/kman.intjssh.4.1.3.

[8] American Psychiatric Association , Neurokognitive Lidelser, Diagnostic and Statistical Manual of Mental Disorders, 2013, 5th edition, American Psychiatric Association, 10.1176/appi.books.9780890425596.

[9] Altarac M. and Saroha E. , Lifetime Prevalence of Learning Disability Among US children, Pediatrics. (2007) 119, no. Supplement_1, S77–S83, 10.1542/peds.2006-2089L, 17272589. 17272589

[10] Bozatlı L. , Aykutlu H. C. , Sivrikaya Giray A. , Ataş T. , Özkan Ç. , Güneydaş Yıldırım B. , and Görker I. , Children at Risk of Specific Learning Disorder: A Study on Prevalence and Risk Factors, Children. (2024) 11, no. 7, 10.3390/children11070759, 39062209. PMC11274916 39062209

[11] Soltani Kouhbanani S. , Arabi S. M. , Zarenezhad S. , and Khosrorad R. , The Effect of Perceptual-Motor Training on Executive Functions in Children With Non-Verbal Learning Disorder, Neuropsychiatric Disease and Treatment. (2020) 16, 1129–1137, 10.2147/NDT.S252662, 32440127. 32440127 PMC7211306

[12] Rochelle K. S. H. and Talcott J. B. , Impaired Balance in Developmental Dyslexia? A Meta-Analysis of the Contending Evidence, Journal of Child Psychology and Psychiatry. (2006) 47, no. 11, 1159–1166, 10.1111/j.1469-7610.2006.01641.x, 17076755. 17076755

[13] Chaix Y. , Albaret J. M. , Brassard C. , Cheuret E. , de Castelnau P. , Benesteau J. , Karsenty C. , and Démonet J. F. , Motor Impairment in Dyslexia: The Influence of Attention Disorders, European Journal of Paediatric Neurology. (2007) 11, no. 6, 368–374, 10.1016/j.ejpn.2007.03.006, 17467315. 17467315

[14] Hussein Z. A. , Abdel-Aty S. A. R. , Elmeniawy G. H. , and Mahgoub E. A. M. , Defects of Motor Performance in Children With Different Types of Specific Learning Disability, Drug Invention Today. (2020) 14, no. 2.

[15] Getchell N. , Pabreja P. , Neeld K. , and Carrio V. , Comparing Children With and Without Dyslexia on the Movement Assessment Battery for Children and the Test of Gross Motor Development, Perceptual and Motor Skills. (2007) 105, no. 1, 207–214, 10.2466/pms.105.1.207-214, 17918566. 17918566

[16] Westendorp M. , Hartman E. , Houwen S. , Smith J. , and Visscher C. , The Relationship Between Gross Motor Skills and Academic Achievement in Children With Learning Disabilities, Research in Developmental Disabilities. (2011) 32, no. 6, 2773–2779, 10.1016/j.ridd.2011.05.032, 21700421. 21700421

[17] Poyraz Findik O. T. , Motor Skills in Children With Specific Learning Disorder: A Controlled Study, Dusunen Adam: Journal of Psychiatry and Neurological Sciences. (2022) 35, no. 2, 101–110, 10.14744/DAJPNS.2022.00181.

[18] Ibrahim S. , Harun D. , Kadar M. , Rasdi H. F. M. , Baharudin S. , and Hui E. J. T. , Motor Performance and Functional Mobility in Children With Specific Learning Disabilities, Medical Journal of Malaysia. (2019) 74, no. 1, 34–39. 30846660

[19] Blanchet M. and Assaiante C. , Specific Learning Disorder in Children and Adolescents, a Scoping Review on Motor Impairments and Their Potential Impacts, Children. (2022) 9, no. 6, 10.3390/children9060892, 35740829. PMC9222033 35740829

[20] Asaseh M. , Fundamental Movements Investigation of Children With Specific Learning Disorder Based on Multivariate Variance Analysis, International Journal of Nonlinear Analysis and Applications, 2020, 11, no. 1, 511–524, 10.22075/ijnaa.2020.4366.

[21] Westendorp M. , Hartman E. , Houwen S. , Huijgen B. C. H. , Smith J. , and Visscher C. , A Longitudinal Study on Gross Motor Development in Children With Learning Disorders, Research in Developmental Disabilities. (2014) 35, no. 2, 357–363, 10.1016/j.ridd.2013.11.018, 24333806. 24333806

[22] Bardid F. , Vannozzi G. , Logan S. W. , Hardy L. L. , and Barnett L. M. , A Hitchhiker′s Guide to Assessing Young People′s Motor Competence: Deciding What Method to Use, Journal of Science and Medicine in Sport. (2019) 22, no. 3, 311–318, 10.1016/j.jsams.2018.08.007, 30166086. 30166086

[23] Robinson L. E. , Stodden D. F. , Barnett L. M. , Lopes V. P. , Logan S. W. , Rodrigues L. P. , and D’Hondt E. , Motor Competence and Its Effect on Positive Developmental Trajectories of Health, Sports Medicine. (2015) 45, no. 9, 1273–1284, 10.1007/s40279-015-0351-6, 26201678. 26201678

[24] Baharudin N. S. , Harun D. , and Kadar M. , An Assessment of the Movement and Function Of Children With Specific Learning Disabilities: A Review of Five Standardised Assessment Tools, Malaysian Journal of Medical Sciences: MJMS. (2020) 27, no. 2, 21–36, 10.21315/mjms2020.27.2.3, 32788838. 32788838 PMC7409574

[25] Burton A. W. and Miller D. E. , Movement Skill Assessment, 1998, Human Kinetics.

[26] Valentini N. C. , Validity and Reliability of the TGMD-2 for Brazilian Children, Journal of Motor Behavior. (2012) 44, no. 4, 275–280, 10.1080/00222895.2012.700967, 22857518. 22857518

[27] Simons J. , Daly D. , Theodorou F. , Caron C. , Simons J. , and Andoniadou E. , Validity and Reliability of the TGMD-2 in 7–10-Year-Old Flemish Children With Intellectual Disability, Adapted Physical Activity Quarterly. (2008) 25, no. 1, 71–82, 10.1123/apaq.25.1.71, 18209245. 18209245

[28] Okuda P. M. M. and Pinheiro F. H. , Motor Performance of Students With Learning Difficulties, Procedia-Social and Behavioral Sciences. (2015) 174, 1330–1338, 10.1016/j.sbspro.2015.01.755.

[29] Nicolson R. I. , Fawcett A. J. , and Dean P. , Developmental Dyslexia: The Cerebellar Deficit Hypothesis, Trends in Neurosciences. (2001) 24, no. 9, 508–511, 10.1016/S0166-2236(00)01896-8. 11506881

[30] Eckert M. A. , Leonard C. M. , Richards T. L. , Aylward E. H. , Thomson J. , and Berninger V. W. , Anatomical Correlates of Dyslexia: Frontal and Cerebellar Findings, Brain. (2003) 126, no. 2, 482–494, 10.1093/brain/awg026, 12538414. 12538414

[31] Diamond A. , Close Interrelation of Motor Development and Cognitive Development and of the Cerebellum and Prefrontal Cortex, Child Development. (2000) 71, no. 1, 44–56, 10.1111/1467-8624.00117, 10836557. 10836557

[32] Nonis K. P. and Jernice T. S. Y. , The Gross Motor Skills of Children With Mild Learning Disabilities, International Journal of Special Education. (2014) 29, no. 2, 92–97.

[33] Anguera J. A. , Reuter-Lorenz P. A. , Willingham D. T. , and Seidler R. D. , Contributions of Spatial Working Memory to Visuomotor Learning, Journal of Cognitive Neuroscience. (2010) 22, no. 9, 1917–1930, 10.1162/jocn.2009.21351, 19803691. 19803691

[34] Buszard T. , Farrow D. , Verswijveren S. J. J. M. , Reid M. , Williams J. , Polman R. , Ling F. C. M. , and Masters R. S. W. , Working Memory Capacity Limits Motor Learning When Implementing Multiple Instructions, Frontiers in Psychology. (2017) 8, 10.3389/fpsyg.2017.01350, 28878701. PMC5572292 28878701

[35] McDougle S. D. and Hillman H. , Motor Working Memory, Trends in Cognitive Sciences. (2026) 30, no. 3, 216–225, 10.1016/j.tics.2025.08.011, 40947323. 40947323 PMC12440371

[36] Roscoe J. T. , Fundamental Research Statistics for the Behavioral Sciences, 1975, 2nd edition, Holt, Rinehart and Winston.

[37] Kaplan B. J. , Dewey D. M. , Crawford S. G. , and Wilson B. N. , The Term Comorbidity Is of Questionable Value in Reference to Developmental Disorders: Data and Theory, Journal of Learning Disabilities. (2001) 34, no. 6, 555–565, 10.1177/002221940103400608, 15503570. 15503570

